# Vitamin A Intake, Serum Vitamin D and Bone Mineral Density: Analysis of the Korea National Health and Nutrition Examination Survey (KNHANES, 2008–2011)

**DOI:** 10.3390/nu7031716

**Published:** 2015-03-10

**Authors:** Nam-Seok Joo, Sung-Won Yang, Byeng Chun Song, Kyung-Jin Yeum

**Affiliations:** 1 Department of Family Practice and Community Health, Ajou University School of Medicine, Suwon 443-781, Korea; E-Mails: jchcmc@daum.net (N.-S.J.); ysw8229@naver.com (S.-W.Y.); 2 Division of Food and Bioscience, College of Biomedical and Health Sciences, Konkuk University, Chungju-si, Chungcheonbuk-do 380-701, Korea; E-Mail: bcsong@kku.ac.kr

**Keywords:** vitamin A, vitamin D, bone mineral density

## Abstract

The association of high vitamin A intake and low bone mineral density (BMD) is still controversial. To determine the association of dietary vitamin A intake and serum 25-hydroxyvitamin D (25(OH)D) concentration with BMD, a total of 6481 subjects (2907 men and 3574 women) aged ≥50 years from the Korean National Health and Nutrition Examination Survey (2008–2011) were divided into groups according to dietary vitamin A intake (tertiles) and serum 25(OH)D (<50, 50–75, >75 nmol/L), and evaluated for BMD after adjusting for relevant variables. Mean dietary vitamin A intakes were 737 and 600 μg RE (Retinol Equivalents) in men and women, respectively. Total hip and femoral neck BMD in men and lumbar spine BMD in women were both positively correlated with dietary vitamin A intake in subjects with serum 25(OH)D >75 nmol/L. Among men with serum 25(OH)D <50 nmol/L, both the top (mean 1353 μg RE) and bottom (mean 218 μg RE) tertiles of dietary vitamin A intake had lower BMD than the middle group (mean 577 μg RE). In this population, BMD was the highest among men and women with serum 25(OH)D = 50–75 nmol/L and that there were no differences in BMD by vitamin A intake in these vitamin D adequate groups. This cross-sectional study indicates that vitamin A intake does not affect bone mineral density as long as the serum 25(OH)D concentration is maintained in the moderate level of 50–75 nmol/L.

## 1. Introduction

Observational studies continuously have reported an association of vitamin A intake or serum retinol concentration with the risk of bone fracture [1,2,3,4]. On the other hand, several studies have found no such association [5,6,7,8]. The classic major role of vitamin A is to maintain epithelial tissues, participate in visual function, spermatogenesis and immune function. Vitamin D also has various biological functions including bone health [9]. Vitamin D increases intestinal absorption of calcium for proper mineralization of bone [10], regulates blood pressure [11,12] and stimulates kidney reabsorption of calcium and phosphate [13]. Persistently increased parathyroid hormone concentrations due to vitamin D deficiency increase bone turnover with a negative bone balance and an increased fracture risk [14]. This leads not only to bone loss but also to increase of bone remodeling which is recognized as an important contributor to bone strength [15].

A modest increase in total fracture risk with high vitamin A and retinol intake was observed in the low vitamin D intake group [16]. In addition, high retinol levels along with vitamin D deficiency were also reported to increase the risk of osteoporosis in postmenopausal women [17]. However, the interrelation between vitamins A and D and their effect on BMD is still unclear, probably due in part to difficulties of obtaining an accurate assessment of vitamin A intake and homeostatic control of serum retinol [18].

Thus, the current study evaluated the relation of vitamin A intake with BMD according to the serum vitamin D concentration utilizing a large population-based dataset, the Korea National Health and Nutrition Examination Survey (KNHANES) from 2008 to 2011.

## 2. Materials and Methods

### 2.1. Study Data and Design

**Figure 1:**
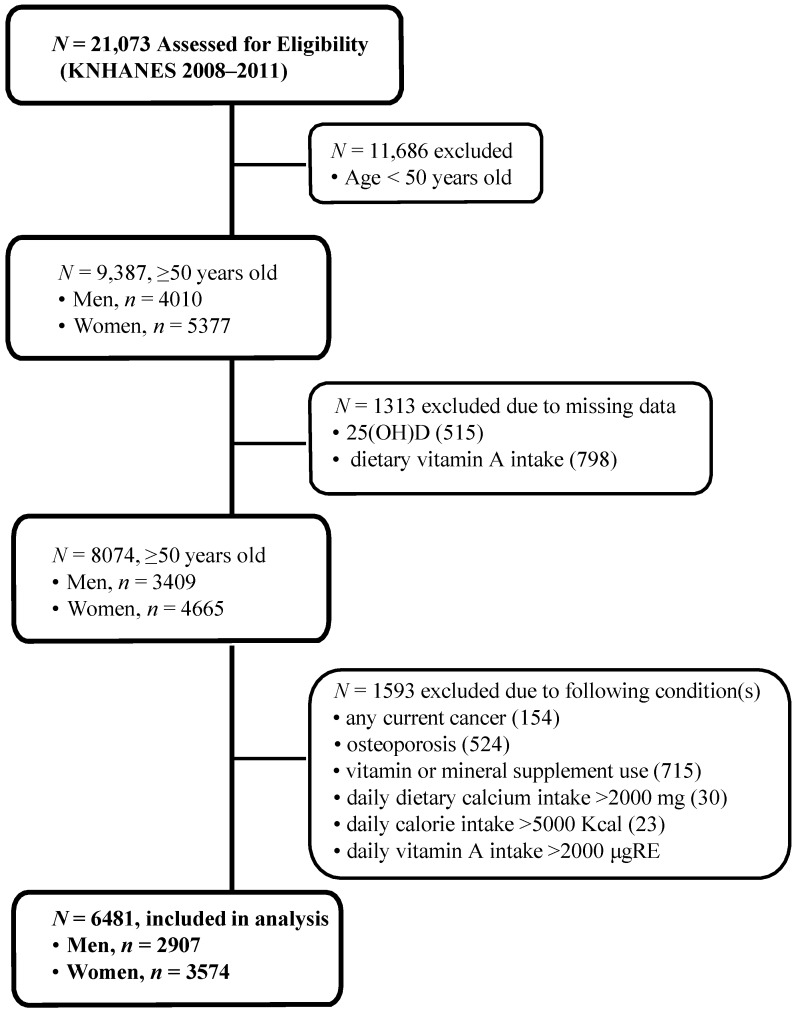
Flow diagram of subject inclusion and exclusion in the Korea National Health and Nutrition Examination Surveys (KNHANES 2008–2011). FRS: Framingham Risk Score.

The Korea National Health and Nutrition Examination Survey (KNHANES), conducted periodically by the Korea Centers for Disease Control and Prevention since 1998, provides comprehensive information about the health status, health behavior, nutritional status and sociodemographic data of 600 national districts in Korea. Four years of data (KNHANES 2008–2011) consisting of a health interview survey, a nutrition survey, and a health examination survey were used in this study. Data about demographic characteristics, diet and health related variables were collected through personal interviews and self-administered questionnaires. Data containing serum 25-hydroxyvitmain D (25(OH)D) concentrations, dietary vitamin A intake and BMD were used in this cross-sectional analysis. From an initial total of 21,073 men and women, 9387 subjects were evaluated (4010 men and 5377 women) aged 50 years and older. From these evaluated subjects, 1313 subjects were excluded because of missing data of serum 25(OH) D (515 subjects) and dietary vitamin A intake (798 subjects). In addition, 1593 subjects were excluded for current cancers (154 subjects), medications that affect calcium metabolism (524 subjects), vitamin or mineral supplement use without documented contents (715 subjects), and overreporting daily dietary calcium intake >2000 mg (30 subjects), calorie intake >5000 kcal (23 subjects), and vitamin A intake >3000 μg RE (149 subjects). Finally, the current study analyzed data of 6481 subjects (2907 men and 3574 women) as shown in Figure 1. All participants provided a written informed consent before the survey. The study protocol was approved by the Institutional Review Board of Ajou University Hospital (MED-MBD-13-328).

### 2.2. Measurements

Blood samples were collected from the antecubital vein of each participant after fasting overnight. The blood samples were properly processed, refrigerated at 2–8 °C and shipped to the Central Testing Institute (NeoDin Medical Institute, Seoul, South Korea). The serum 25(OH)D concentration was measured with a radioimmunoassay (RIA) kit (DiaSorin Inc., Stillwater, MN, USA) using a γ-counter (1470 Wizard; PerkinElmer, Turku, Finland). Serum 25(OH)D was measured in one institute. Quality control was conducted every other week throughout the analysis period to minimize the analytical variation. Serum 25(OH)D concentrations were stratified into three groups: vitamin D deficiency (<50 nmol/L), vitamin D insufficiency (50–75 nmol/L) and vitamin D sufficiency (>75 nmol/L) according to the estimates of optimal vitamin D status reported by Dawson-Hughes *et al.* [19].

Nutrient intakes including those of total calorie and vitamin A were assessed with a 24 h dietary recall questionnaire administered by a trained dietician. The results were calculated using the Food Composition Table developed by the National Rural Resources Development Institute (7th revision) [20]. Contents of dietary supplements were not documented in the KNHANES.

The bone mineral density (BMD) (g/cm^2^) measured at the lumbar spine and neck of the femur (femoral neck BMD) were conducted using dual-energy X-ray absorptiometry (DXA, DISCOVERY-W fan-beam densitometer, Hologic Inc, Bedford, MA, USA) with the CVs of 1.9% for the L1–4 spine and 2.5% for the femoral neck.

### 2.3. Lifestyle Questionnaires

Physical activity was assessed by a questionnaire and categorized as “yes” or “no” with “yes” meaning >30 min of moderate physical activity three or more times in the last week in which the subject was tired compared to ordinary levels. Current smokers were defined as those who had smoked more than five packs of cigarettes during their life and were currently smoking; ex-smokers were smokers who had smoked in the past but had quit; non-smokers had no history of smoking. Regular alcohol drinkers were those who currently drank alcohol more than one time per month and nondrinkers were all others. Job was classified into two groups: in-door workers and out-door workers. The in-door workers included managers, professionals and related workers, clerical office workers, service workers and sales workers. Unemployed subjects were classified as in-door workers considering their limited out-door activities. The out-door workers included skilled agricultural, forestry and fishery workers. Education also was assessed by a self-administered questionnaire (elementary, middle, high, university or college). Women were classified into menopausal status, oral contraceptive use and hormone replacement therapy.

### 2.4. Data Analysis

The complex sample analysis was used for the KNHANES data for the assessment of all values following the statistics guidance of the Korea Centers for Disease Control and Prevention. General characteristics for both genders were presented including age, body mass index, waist circumference, daily dietary total energy intake, daily dietary calcium intake, daily dietary vitamin A intake, serum 25(OH)D concentrations and BMD (total hip, femoral neck and lumbar spine BMD). To evaluate the association between dietary vitamin A and each BMD (total hip, femoral neck and lumbar spine BMD), a partial correlation analysis was conducted in both genders according to the classification of serum 25(OH)D concentration (<50 nmol/L, 50–75 nmol/L, and >75 nmol/L) after adjusting for age, BMI, job, education, alcohol intake, smoking status and moderate physical activity, total energy intake, dietary calcium intake and menopause, oral contraceptive use, hormone replacement therapy in case of women. To determine further the association between dietary vitamin A and each BMD, dietary vitamin A intakes were divided into tertiles in each gender. In these dietary vitamin A categories, the differences between all BMD were determined by the serum 25(OH)D classification using the ANCOVA test after adjusting for age, BMI, job, education, alcohol intake, smoking status and moderate physical activity, total energy intake, dietary calcium intake and menopause, oral contraceptive use, hormone replacement therapy in case of women. All *p* values were *p* for trend used to assess the significance of all analyses, and *p* < 0.05 was considered significant. Data were analyzed using SPSS 18.0 (SPSS Inc., Chicago, IL, USA).

## 3. Results

### 3.1. Subjects

Mean ages of men (*n =* 2907) and women (*n =* 3574) in this study were 61.0 and 62.4 years old, respectively. Dietary vitamin A intakes were 737 μg RE and 600 μg RE, respectively. Their dietary calcium intakes were 537 mg/day and 413 mg/day and serum 25(OH)D were 52 nmol/L and 45 nmol/L, respectively (Table 1).

**Table 1:** General characteristics of study subjects by gender.

Variables	Men (*n =* 2907)	Women (*n =* 3574)
Age (year)	61.0 (0.2)	62.4 (0.2)
BMI (kg/m^2^)	23.8 (0.1)	24.3 (0.1)
Waist circumference (cm)	85.5 (0.2)	82.6 (0.2)
Total energy intake (kcal/day)	2138.2 (19.4)	1542.5 (14.6)
Dietary calcium intake (mg/day)	536.7 (7.2)	413.1 (6.1)
Dietary vitamin A intake (μg/RE)	736.9 (14.7)	600.3 (12.8)
25(OH)D (nmol/L)	52.1 (0.6)	45.0 (0.5)
THBMD (g/cm^2^)	0.931 (0.003)	0.784 (0.003)
FNBMD(g/cm^2^)	0.755 (0.003)	0.635 (0.002)
LSBMD(g/cm^2^)	0.947 (0.004)	0.822 (0.003)

Data are expressed as mean (standard error); BMI, body mass index; 25(OH)D, serum 25-hydroxyvitamin D concentration; THBMD, total hip bone mineral density; FNBMD, femoral neck bone mineral density; LSBMD, lumbar spine bone mineral density.

### 3.2. Correlation Between Dietary Vitamin A and BMD by the Serum 25(OH)D Concentration

Each BMD (total hip, femoral neck and lumbar spins) was compared with dietary vitamin A intakes according to the serum 25(OH)D status (<50, 50–75, >75 nmol/L) using partial correlation after adjusting for age, BMI, job, education, alcohol intake, smoking status and moderate physical activity, total energy intake, dietary calcium intake and menopause, oral contraceptive use, hormone replacement therapy. Among the men who had serum 25(OH)D concentrations higher than 75 nmol/L, total hip BMD and femoral neck BMD had positive correlations with dietary vitamin A. In women, lumbar spine BMD was positively correlated with dietary vitamin A intake in the serum 25(OH)D >75 nmol/L. Among the men with vitamin D deficiency (<50 nmol/L), dietary vitamin A intake and each BMD showed insignificant but negative correlation (Table 2).

**Table 2:** Partial correlation of dietary vitamin A and bone mineral density by serum 25-hydroxyvitamin D concentration.

Serum 25-Hydroxyvitamin D (nmol/L)						
	Men			Women		
BMD	<50	50~75	>75	<50	50~75	>75
THBMD	−0.044	0.024	0.129 *	0.006	0.009	0.099
FNBMD	−0.035	0.053	0.130 *	0.011	0.021	0.107
LSBMD	−0.036	0.012	0.024	0.009	−0.027	0.159 *

Values represent partial correlation coefficient after adjusting for age, body mass index, job, education, smoking, alcohol intake, moderate physical activity, total energy intake, dietary calcium intake and menopause, oral contraceptives, hormone replacement therapy in case of women. THBMD, total hip bone mineral density; FNBMD, femur neck bone mineral density; LSBMD, lumbar spine bone mineral density; * *p* < 0.05.

### 3.3. Vitamin A Intake and BMD by the Serum 25(OH)D Concentration

**Figure 2:**
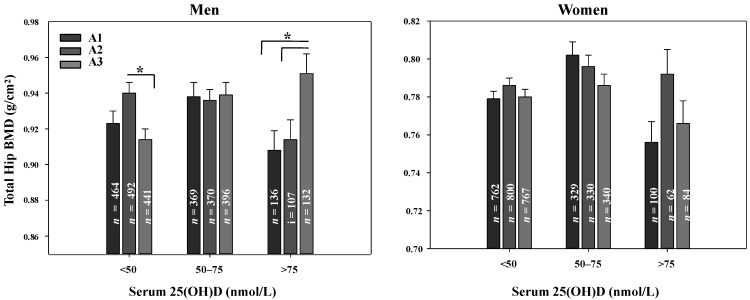

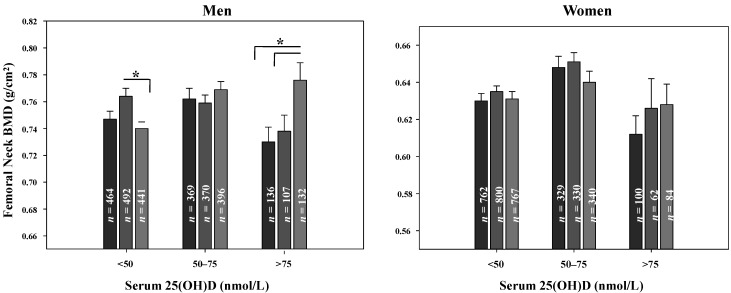
The bone mineral density of total hip and femur neck by serum 25-hydroxyvitamin D concentration and dietary vitamin A intake. Three bars represent tertiles of vitamin A intakes (A1, mean 217.8; A2, mean 577.4; A3, mean 1353.4 μg RE) for men and (A1, mean 156.4; A2, mean 449.6; A3, mean 1163.6 μg RE) for women. BMD, bone mineral density; serum 25(OH)D, serum 25-hydroxyvitamin D concentration. Data are adjusted for age, BMI, total calorie intake, calcium intake, job education, smoking, alcohol, moderate physical activity. *p* for trend analyzed by general linear model in complex data analysis.

We categorized dietary vitamin A intakes into tertiles and compared these with each BMD by the serum 25(OH)D concentrations (<50, 50–75, >75 nmol/L) after adjusting for relevant variables. When the interaction of two independent variables, vitamins A tertile and D, was determined after adjusting for age, BMI, job education, smoking alcohol intake, physical activity, energy intake, dietary calcium intake and menopause, oral contraceptives, hormone replacement therapy in case of women, there was significant interaction in the femoral neck of men (*p =* 0.038), but not in the other sites (total hip, *p =* 0.067; lumbar spine, *p =* 0.395). Women did not show any interaction. Among men with serum 25(OH)D <50 nmol/L, both the top (mean 1353 μg RE; 801.3–2990.4 μg RE) and bottom (mean 218 μg RE; ≤378.6 μg RE) tertiles of dietary vitamin A intake had a lower BMD than the middle group (mean 577 μg RE; 378.7~801.2). In this population, BMD was the highest among men and women with serum 25(OH)D 50–75 nmol/L, and there were no differences in vitamin A intake in these vitamin D adequate groups (Figure 2).

## 4. Discussion

In this cross-sectional study, we found that an adequate serum vitamin D concentration was important for bone health and that the vitamin A intake was associated with BMD only in vitamin D deficient men (<50 nmol/L). As long as the serum vitamin D was adequate (50–75 nmol/L), vitamin A intake did not affect the BMD. When serum vitamin D was sufficient (>75 nmol/L), a positive correlation between vitamin A intake and BMD was found. These findings may have significant implications in this population, which have a very low vitamin D intake in general due to the low intakes of vitamin D-rich foods and limited availability of vitamin D-fortified foods [21]. The recommended dietary allowance for vitamin A is 700 µg RE/day for males and 600 µg RE/day for females and for calcium it is both 700 mg/day for males and females aged ≥50 years according to the Dietary Reference Intakes for Koreans 2010 [22]. In this study, 60% of males and 63.1% of females consumed less than the recommended vitamin A amount, and 78.2% of males and 87% of females were under the recommended intake of calcium for Koreans. In addition, 48% of men and 65% of women aged ≥50 years had less than 50 nmol/L of serum 25(OH)D.

In an animal study, marginal dietary vitamin A intake (0.35 μg retinol/g diet) was reported to affect trabecular bone, and moderate vitamin A supplementation over the lifetime would not increase the risk of age-related bone fracture [23]. In addition, vitamin A analogue treatments reported to have a beneficial effect against fracture [24]. On the other hand, previous human studies indicated that a high preformed vitamin A intake (1500–2000 μg/day) or high serum retinol concentration was correlated with low BMD or increased risk of fractures [1,2]. In addition, serum retinyl ester concentration was marginally associated with osteoporosis [25]. In older women, higher serum retinol in a normal range was reported to maintain BMD, whereas too much retinyl ester intake was associated with decreased BMD [26]. Proposed mechanisms for such negative effects of the excess vitamin A on BMD are a reduction of bone-forming surfaces at the subperiosteal site in adult rodents [27], weakening of bones and increased expression of hypoxia-associated genes [28]. In addition, retinoic acid increases proliferation of human osteoclast progenitors. It can inhibit the receptor activator of nuclear factor kappa B (RANK)-stimulated osteoclast differentiation by suppressing RANK [29], and inhibit Nuclear factor of activated T-cells, cytoplasmic 1 (NFATc1) expression and osteoclast differentiation [30], which is associated with bone fragility and fractures.

The current study reports that an adequate vitamin D status is associated with higher BMD and the association of vitamin A with BMD appears to vary by vitamin D status in men. We cannot overlook a possibility of bias evaluating the vitamin A intake through, for example, milk [31], butter [32] and liver [33,34], since these foods can be related with low bone mass or osteoporosis risk due to an environmental toxin or other negative effect on bone. In addition, there is a potential for misclassification of vitamin A intake assessed by a 24-hour dietary recall questionnaire. However, previous studies indicating that vitamin D insufficiency together with high serum concentrations of vitamin A increases the risk for osteoporosis in postmenopausal women [35] is in line with this current study.

Although it is hard to confirm whether serum 25(OH)D has a property preventing the negative effect of vitamin A on BMD, a previous study has indicated that an excess of vitamin A reduces the efficacy of vitamin D and potentiates the vitamin D deficiency-insufficiency effect [36]. It is also possible that vitamin A inhibits the favorable effect of vitamin D on bone mineral density by competing with its receptors [37]. Therefore, an adequate bone mineral density may depend on an optimal ratio of retinol to 25(OH)D concentration as a consequence of a suitable ratio of vitamins A to D intake [38]. Theoretically, under a vitamin D sufficient status, the negative effect of a high vitamin A intake on BMD can be prevented if the action of vitamin D via VDR/RXR in competition with RAR/RXR is more prominent. In our study, an approximately two-fold higher intake of vitamin A (top tertile, mean 1353 μg RE) compared to the recommendation (700 μg RE) resulted in a low BMD in vitamin D deficient men. Therefore, an avoidance of a high intake of vitamin A may be recommended in a vitamin D deficient population. On the other hand, if a serum concentration of up to 50 nmol/L of 25(OH)D can be achieved, relatively high dietary vitamin A is not a risk anymore and can be expected to exert synergy for BMD.

Due to the limitation of the cross-sectional nature of this study, the cause of low BMD in men with high vitamin A intake along with low vitamin D status cannot be determined. In addition, a very low proportion of women with vitamin D sufficiency (6.8%) in this population made it difficult to evaluate the synergistic interaction between sufficient vitamins A and D for BMD. The negative effect of excessive vitamin A intake and vitamin D deficiency on BMD found in men was also not evident in women. Even though there were enough women in the vitamin D deficient group (65.2%), relatively moderate vitamin A intake in the top tertile of the vitamin A intake group in women (mean 1163 μg RE) compared to that of men (1353 μg RE) may not be high enough to have negative effect on BMD. The current study has another limitation that the 25(OH)D data were not adjusted for some confounders such as overall sun-exposure and season due to the inaccessibility. However these confounders for vitamin D status may not be substantial factors in this study since blood samples were collected year-round and similar numbers of subjects were measured in each season for the KHNAES. Finally, we should not overlook the fact that the localized increase of BMD such as in the femoral neck but not in the lumbar spine could be artifactual as reported recently [39].

## 5. Conclusions

We found that adequate vitamin D status is associated with higher BMD and that the association between vitamin A and bone mineral density can be negligible in populations having a serum level of vitamin D ≥50 nmol/L. Vitamin A intake exceeding recommendation may have a negative implication on bone health only for a population with vitamin D deficiency.
